# Current Treatments, Emerging Therapeutics, and Natural Remedies for Inflammatory Bowel Disease

**DOI:** 10.3390/molecules29163954

**Published:** 2024-08-21

**Authors:** Karma Yeshi, Tenzin Jamtsho, Phurpa Wangchuk

**Affiliations:** 1 College of Public Health, Medical, and Veterinary Sciences (CPHMVS), James Cook University, Building E4, McGregor Rd, Smithfield, Cairns, QLD 4878, Australia; karma.yeshi@jcu.edu.au; 2 Australian Institute of Tropical Health and Medicine (AITHM), James Cook University, Building E4, McGregor Rd, Smithfield, Cairns, QLD 4878, Australia

**Keywords:** small molecules, biologics, novel IBD treatments, natural products, artificial intelligence

## Abstract

Inflammatory bowel disease (IBD) is a chronic, lifelong disorder characterized by inflammation of the gastrointestinal (GI) tract. The exact etiology of IBD remains incompletely understood due to its multifaceted nature, which includes genetic predisposition, environmental factors, and host immune response dysfunction. Currently, there is no cure for IBD. This review discusses the available treatment options and the challenges they present. Importantly, we examine emerging therapeutics, such as biologics and immunomodulators, that offer targeted treatment strategies for IBD. While many IBD patients do not respond adequately to most biologics, recent clinical trials combining biologics with small-molecule drugs (SMDs) have provided new insights into improving the IBD treatment landscape. Furthermore, numerous novel and specific therapeutic targets have been identified. The high cost of IBD drugs poses a significant barrier to treatment, but this challenge may be alleviated with the development of more affordable biosimilars. Additionally, emerging point-of-care protein biomarkers from serum and plasma are showing potential for enhancing the precision of IBD diagnosis and prognosis. Several natural products (NPs), including crude extracts, small molecules, and peptides, have demonstrated promising anti-inflammatory activity in high-throughput screening (HTS) systems and advanced artificial intelligence (AI)-assisted platforms, such as molecular docking and ADMET prediction. These platforms are advancing the search for alternative IBD therapies derived from natural sources, potentially leading to more affordable and safer treatment options with fewer side effects.

## 1. Introduction

Inflammatory bowel disease (IBD) is a chronic, recurring inflammatory condition of the gastrointestinal (GI) tract with a multifaceted cause, typically manifesting in adolescence or early adulthood [1]. Inflammatory bowel diseases have significantly impacted global health over the past three decades, with a 47% increase in incidence and a 69% rise in mortality worldwide [2]. Inflammatory bowel diseases comprise two subtypes, ulcerative colitis (UC) and Crohn’s disease (CD), with UC specifically affecting the mucosal layer of the colon and rectum. In contrast, CD is more complex, affecting the entire GI tract, most commonly the ileum, and involves full-thickness inflammation [3]. The extent of inflammation in UC varies, ranging from proctitis (inflammation of the rectum) to left-sided or distal colitis or extensive colitis (pancolitis) (3). In CD, inflammation is non-contiguous, affecting the colon, the ileum, or both (ileocolonic). Ulcerative colitis is a T-helper cell type 2 (Th2)-driven disease mediated by interleukin (IL)-5 and IL-13, whereas CD involves a Th1 response with interferon (IFN)-γ and IL-2 cytokines [4]. 

Clinically, IBD is a heterogeneous group of diseases with varying pathological symptoms. Thus, differentiating UC from CD is difficult in almost 5–15% of IBD patients [3], suggesting that the pathophysiology of IBD is multifaceted. The common IBD symptoms include loss of appetite and weight, diarrhoea, abdominal pain, fatigue, anaemia, fever, or night sweats. 

The exact aetiology of IBD is still yet to be ascertained, and as a result, there is currently no cure for IBD. Several studies have attributed the cause of IBD to a combination of multiple factors, including genetic susceptibility, immune response dysfunction, gut microbial dysbiosis, and environmental factors [5]. Recently, Boaz et al. (2022) [6] investigated the potential risk of developing IBD due to family history by comparing 35 familial and 88 sporadic IBD patients. The study determined that familial IBD has a stronger association with the early onset of IBD with more adverse phenotypes. Similarly, patients with familial CD showed more adverse clinical outcomes than those with sporadic CD [7]. Additionally, altered gut microbiota and resultant metabolites may also be implicated in IBD pathogenesis, including colorectal cancer. For instance, a genetically susceptible individual has a dysregulated mucosal immune response to commensal gut flora (microbial dysbiosis) [8]. On the other hand, Brand et al. [9] showed that IBD patients share gut microbiome signatures with their healthy co-twins. 

Altered gut microbiota and their metabolites play a vital role in IBD pathogenesis. Liu et al. (2022) [10] analysed the gut bacterial diversity between the genetic variant mice carrying Atg16L1T300A and their wild type; genetic variant mice had more abundance of bacteria associated with IBD (e.g., *Tyzzerella*, *Mucispirillum*, *Ruminococcaceae*, and *Cyanobacteria*). Moreover, there were reduced mucin secretion and bacteria associated with mucin production (*Akkermansia*) compared to the wild type. The result suggests that altered microbiota may increase the risk of developing CD among carriers of this genetic variant. 

Due to the complexity of the disease, currently available treatments can only induce remission among the patients, and unfortunately, many patients relapse at some time point [11]. Therefore, there is an urgent need for better treatment options for IBD patients or, otherwise, a cure. Given the numerous side effects of current therapies, alternative treatments from natural products, such as medicinal herbs and helminths, are highly sought after due to their reduced side effects. This review explores potential natural product solutions compared to existing treatments to improve the lives of IBD patients. Information on IBD and natural products was retrieved through a comprehensive literature search using PubMed, Scopus, Google Scholar, Web of Science, and MEDLINE Ovid online databases, with suitable keywords, including ‘BD’, ‘ulcerative colitis’, ‘Crohn’s disease’, ‘IBD therapies’, ‘challenges’, ‘side effects’, ‘biologics’, ‘immunomodulators’, ‘helminths’, ‘microbes’, ‘small molecules’, ‘emerging therapeutics for IBD’, ‘traditional treatments’, ‘IBD drugs’, ‘clinical trial’, ‘natural products’, ‘anti-inflammatory’, ‘artificial intelligence (AI)’, and ‘AI in drug discovery’.

## 2. Existing IBD Treatments and Challenges 

IBD treatment initially relied on corticosteroids, aminosalicylates (ASA), and immunosuppressants for many years. Olsalazine, balsalazide, and sulfasalazine are common oral aminosalicylates for treating mild to moderately active UC (Table 1). However, they are associated with side effects, including cardio- and hepatorenal toxicity and sexual dysfunction [12]. Corticosteroids can reduce colonic inflammation via downregulating the nuclear factor kappa B (NF-κB) pathway [13]. Budesonide (corticosteroid) can treat both UC and CD, but it is unsuitable for short-term treatment due to its low bioavailability and first-pass effect when taken orally [12]. Since prolonged dependence on corticosteroids has numerous side effects (Table 1), IBD patients who are refractory to or rely on corticosteroids use immunosuppressant drugs, such as methotrexate (MTX) and 6-mercaptopurine (6-MP). They are cheaper and accessible to take orally [12], but MTX is toxic to bone marrow and liver, and it is not suitable for pregnant patients as they are also toxic to embryos [12,14]. Cyclosporine is considered less effective than tacrolimus, especially in oral form, due to its lower absorption in the colon [12,15]. These conventional therapies have numerous side effects and limited efficacy; new effective drugs with fewer side effects are required. 

The advent of biologics, such as anti-TNF agents, has improved the treatment strategy for IBD, as they are more specific to the disease target than conventional therapies. Four TNF inhibitors are currently available for treating IBD: infliximab and adalimumab (for UC and CD), certolizumab (CD only), and golimumab (UC only) (Table 1). Anti-TNF antibodies neutralise secretory TNF (s-TNF) and transmembrane TNF (tm-TNF) from binding to their receptors, thus alleviating inflammation [12]. They either induce apoptosis of TNF-producing cells or block leucocyte infiltrations by downregulating cell adhesion proteins (such as e-selectin, ICAM-1, and VCAM-1) [17]. Although TNF inhibitors are one of the preferred therapies for IBD, their repeated use may induce immunogenicity [18]. Moreover, during their initial treatment, up to 30% of patients do not respond adequately (primary non-responder), and 40% relapse during treatment (secondary non-response) [19]. Thus, stratification of subjects at risk of developing immunogenicity and identifying non-responders is essential before giving/choosing anti-TNF therapies. 

The treatment option for IBD has further widened with the approvals of anti-integrins (vedolizumab and natalizumab), Janus kinase (JAK) inhibitors (tofacitinib, filgotinib, and upadacitinib), and anti-p19 antibodies (ustekinumab and risankizumab) (Table 1). Chu et al. (2023) [20] conducted a network meta-analysis on the efficacy and safety of anti-integrin antibodies against UC. They found that vedolizumab had the highest efficacy in achieving and maintaining clinical remission. While infliximab showed the highest efficacy for endoscopic improvement, guselkumab and ustekinumab exhibited the lowest risks for recurrence and adverse events for UC, respectively [20]. 

Could combining multiple biologics be an alternative therapy to maximise efficacy with fewer side effects? Several studies have tried combination therapies (CoT) of biologics or biologics with SMD (e.g., anti-TNF + anti-integrins) against IBD. A recent phase 2a VEGA study by Sands et al. (2022) [21] compared a CoT using guselkumab plus golimumab over their monotherapy in adults with moderate to severe active UC. Patients who received CoT showed a significantly higher clinical response (83.1%) than those who received monotherapy with guselkumab (74.6%) or golimumab (61.1%). Kwaspisz et al. (2021) [22] and Ahmed et al. (2022) [23] also showed similar results with an anti-TNF or vedolizumab with ustekinumab as an ideal combination therapy for IBD besides minor adverse events such as *Salmonella gastroenteritis* and *Clostridium difficile* infections. Although more studies will be required, promising results such as mucosal healing [24,25] and safety profiles from CoT [26,27] have brought new hope for IBD patients. Biosimilars, for example, CT-P13 and exemption for infliximab and adalimumab, respectively, are already in the market, which has eased the affordability of treatment for many IBD patients. A study conducted by Schreiber et al. (2021) [28] obtained similar efficacy between infliximab and its biosimilar, CT-P13. Despite similar effectiveness and safety, the exemption cost is one-fifth of adalimumab [12].

## 3. Therapeutic Drugs for IBD in the Pipeline

The advent of new biological and small-molecule therapies has made significant progress in the treatment landscape of IBD, and many more are in the pipeline (Table 2). Janus kinase (JAK) inhibitors, immunosuppressants, and anti-trafficking molecules are a few examples. Compared to biologics, SMD is cheaper and has a shorter half-life and low immunogenicity. JAK is a non-receptor tyrosine-protein kinase that mediates cytokine signalling, and there are four types: JAK1, JAK2, JAK3, and tyrosine kinase 2 (TYK2) [12]. As intracellular signal mediators interact and work in pairs, JAK interacts with signal transducers and activators of transcription [29], forming the JAK-STAT signalling pathway, which transmits inflammatory signals to the nucleus. Blocking this pathway cuts the inflammatory signals reaching the nucleus, thereby reducing the synthesis of downstream inflammatory cytokines and inflammation [30]. Tofacitinib, upadacitinib, and filgotinib are a few examples of recently approved JAK inhibitors for UC. 

Tofacitinib, a JAK inhibitor, has demonstrated good CD tolerance in phase II trials [31]. Upadacitinib, a second-generation JAK inhibitor, has demonstrated better selectivity for JAK1 and JAK2 than tofacitinib for UC and is now in phase III trials for CD [32]. It has shown superior endoscopic improvements but is associated with adverse effects like pneumonia, nasopharyngitis, gastroenteritis, and malignancies [33,34]. Filgotinib, another JAK1 inhibitor, is in phase III trials for CD [35]. Deucravacitinib, a TYK2 inhibitor, is in phase II trials for both UC and CD [31]. Mongersen, a Smad7 antisense oligonucleotide, can restore TGF-β1-Smad signalling and is in phase II trials for UC [31]. 

Sphingosine-1-phosphate (SI1P) modulators are another new therapeutic drug for IBD. S1P is a lysophospholipid signalling metabolite which binds to G-protein-coupled receptors (S1PR1-5) on T cells [12], promoting differentiation, migration and proliferation of lymphocytes. S1P modulators block the S1P pathway, as both UC and CD are due to lymphocyte recruitment into the GI tract. Ozanimod, an oral S1P/S1P5 receptor agonist, is in phase III trials for CD [36]. Estrasimod, another S1P inhibitor, is under development and has shown better clinical remission rates in phase III trials for UC compared to placebo, with no reported deaths or malignancies [37]. 

Cytokine inhibitors such as anti-IL12/23 agents block the p35 and p40 subunits of IL-12 and the p19 and p40 subunits of IL-23, essential for differentiating CD4+ T cells [38]. Interleukin (IL)-12/IL-23 inhibitors (e.g., ustekinumab) prevent the interaction of these cytokines with their receptors, subsequently blocking the IL-12/IL-23 signalling to prevent further activation of Th1/Th17 cells involved in the pathogenesis of CD [12,38]. 

More IL-12/IL-23 inhibitors and anti-integrin/anti-adhesion agents are undergoing clinical assessment for their efficacy and safety in treating IBD (Table 2) [39,40,41,42,43]. Generally, biologics are considered better than SMD as biologics are targeted treatment and could reduce the hospitalisation rate and produce improved long-term effects [44]. However, they are expensive, can produce life-threatening side effects, and not all patients can afford these biologics. Thus, a significant proportion of patients still require surgical treatment. 

One of the challenges in IBD drug clinical trials is the need for standardised endpoints. For instance, determining appropriate clinical endpoints (e.g., mucosal healing, clinical remission) that are universally accepted and meaningful is challenging. Additionally, maintaining patient participation over long trial periods can be difficult due to the chronic nature of the disease and the potential side effects of the treatment, as mentioned above. IBD trials often show high placebo response rates [45], which can obscure the actual effectiveness of the investigational drug. Despite these challenges, engaging patients and advocacy groups to ensure trial designs meet patient needs may help improve recruitment and retention. Thus, a collaborative and multifaceted approach, combining scientific, regulatory, and patient-centred strategies, should be adopted to expedite the IBD drug development process. 

**Table 2:** Therapeutic drugs and targets for treating IBD in the pipeline.

Types of Treatment	Drugs	Route of Administration	Drug Target	Clinical Trial Phase	References
Anti-adhesion/anti-trafficking molecules	Abrilumab (AMG181)	SC	α4β7-integrin	CD: II; UC: II	[31]
	AJM 347	Oral	α4β7-integrin	UC: I/II	[31]
	Alicaforsen	Oral	ICAM-1 mRNA	CD: III; UC: II	[31]
	Carotegrast methyl (AJM 300)	Oral	α4-integrin	UC: III	[46]
	Etrolizumab	IV, SC	α4β7, αEβ7, and β7-integrins	CD: III; UC: III	[46,47,48]
	GSK1605786A	Oral	CCR9	CD: III	[31]
	Natalizumab	IV	α4-integrin	CD: III	[31]
	Ontamalimab (PF-00547659)	SC	MAdCAM	CD: II; UC: II	[31]
	Ontamalimab (SHP647)	SC	MAdCAM-1	CD: III; UC: III	[31]
	PN-943	Oral	α4β7-integrin (gut restricted)	UC: II	[31]
	PTG-100	Oral	α4β7-integrin	UC: 11a	[31]
	Vedolizumab SC	SC	α4β7-integrin	CD: III; UC: III	[49]
Anti-TNF	CT-P13	SC	TNF	CD: III; UC: III	[31]
	OPRX-106	Oral	TNF	UC: II	[31]
IL-10 fusion biologic	AAMT-101	Oral	IL-10	UC: Ia	[31]
IL-12/IL-23 inhibitors	Brazikumab	IV, SC	p19 subunit of IL-23	CD: I; UC: I	[42]
	Guselkumab	SC	p19 subunit of IL-23	CD: III; UC: III	[43]
	Mirikizumab	IV, SC	p19 subunit of IL-23	CD: III; UC: III	[40,41]
	Risankizumab	IV	Cytochrome p450	CD: I; UC: I	[31]
	Risankizumab	IV, SC	p19 subunit of IL-23	UC: III	[39,41]
IL-36 inhibitor	Spesolimab	IV	IL-36R	CD: II; UC: III	[31]
Immunosuppressants	GSK2831781	IV	LAG3	UC: II	[31]
	Ravagalimab (ABBV-323)	IV, SC	CD40	UC: IIa	[31]
JAK inhibitors	Brepocitinib (PF-06700841)	Oral	TYK2/JAK1	CD: IIa; UC: IIb	[50]
	Deucravacitinib (BMS-986165)	Oral	TYK2	CD: II; UC: II	[31]
	Filgotinib	Oral	JAK1	CD: III	[35]
	Ivarmacitinib	Oral	JAK1	UC: II	[31]
	Izencitinib (TD-1473)	Oral	Gut-selective pan-JAK	UC: III	[31]
	Peficitinib	Oral	JAK3	UC: IIb	[31]
	Ritlecitinib (PF-06651600)	Oral	JAK3/TEC kinase	CD: II; UC: II	[50]
	SHR-0302	Oral	JAK1	CD: II; UC: II	[31]
	Tofacitinib	Oral	JAK1/JAK3	CD: II	[31]
	Upadacitinib	Oral	JAK1	CD: III	[33,34]
PDE4 inhibitor	Apremilast	Oral	PDE4	UC: II	[51]
S1P receptor modulators	Amiselimod (MT-1303)	Oral	S1PR1,5	CD: II; UC: II	[31]
	CBP-307	Oral	S1PR1	UC: II	[31]
	Etrasimod	Oral	S1PR1/S1PR4/S1PR5	CD: III; UC: III	[31]
	Ozanimod	Oral	S1PR1/S1PR5	CD: III	[36]
Smad7 antisense oligonucleotide	Laquinimod	Oral	NF-κB	CD: IIa	[31]
	Mongersen (GED-0301)	Oral	Smad7	UC: II	[31]
	Thalidomide	Oral	CRBN	CD: II; Pediatric IBD: III	[31]
Spore-based microbiome	SER-287	Oral	*Firmicutes*	UC: Ib	[52]
TLI1A agonist	PF-06480605	SC	TL1A/TNFSF15	UC: II	[31]
TLR9 agonist	Cobitolimod	Topical (enema)	TLR9	UC: III	[31]
NP-derived	Curcumin and artesunate	Oral	NA	CD: IIa	[31]
	Mastiha	Oral	NA	UC: II	[31]
	Saffron extract	Oral	NA	UC: II	[31]
	*Trichuris suis* ova (TSO)	Oral	NA	UC: II	[31]

CD: Crohn’s disease; CD: cluster of differentiation; CCR9: chemokine receptor-9; CRBN: cereblon; IL: interleukin; IV: intravenous; SC: sub-cutaneous; ICAM-1: intercellular adhesion molecule-1; JAK: Janus kinase; LAG3: lymphocyte activation gene 3; MAdCAM-1: mucosal addressin cell adhesion molecule 1; mRNA: messenger RNA; NA: not available; NF-κB: nuclear factor kappa B; PDE4: phosphodiesterase 4 inhibitor; S1P: sphingosine-1-phosphate; Smad: mothers against decapentaplegic homolog; TEC: Tec kinase; TLRs: toll-like receptors; TNFSF: TNF receptor superfamily; TYK: tyrosine kinase; UC: ulcerative colitis. All details regarding the clinical trials of IBD drugs currently in development were obtained from www.clinicaltrials.gov (accessed on 30 June 2024) [31].

## 4. Natural Products as Potential Anti-Inflammatories for Treating IBD 

### 4.1. Plants—Higher Plants, Fungi, and Medicinal Plants 

**Figure 1:**
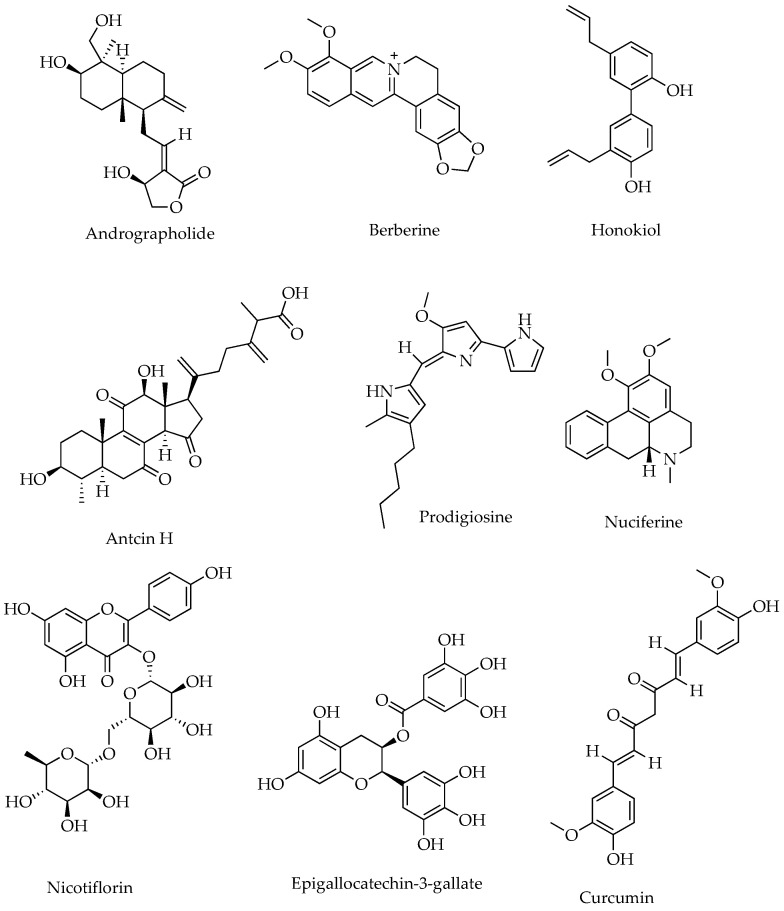
Representative phytochemical structures of anti-inflammatory SMs.

Natural products (NPs) and their derivatives have been a promising pool for discovering therapeutic leads, including anti-inflammatories. Medicinal plants and their traditional formularies have shown protection against colonic inflammation by restoring epithelial tight junctions, increasing mucin secretion, preventing luminal microbial dysbiosis, and reducing oxidative stress in the gut [53]. More than 79781 NP-derived small molecules have been registered in the anti-inflammatory compound database (AICD), freely accessible at https://956023.ichengyun.net/AICD/index.php (accessed on 22 July 2024). Moreover, as many as 28 randomised clinical trials (RCTs) on 18 herbs, including *Curcuma longa* (e.g., curcumin), have shown promising results against IBD [54]. For instance, curcumin isolated from *C. longa* in combination with artesunate (a derivative of artemisinin isolated from *Artemisia annua*) is currently under phase II clinical trial for CD [31]. Berberine, the main bioactive component in many plants, including the Chinese medicinal herb *Coptis chinensis,* reduced the recurrence rate of UC remission; however, it was withdrawn from phase IV clinical trials due to lack of funding [31]. Other plant-derived IBD clinical therapeutic leads are epigallocatechin-3-gallate (isolated from *Camellia sinensis*) and triptolide (isolated from *Tripterygium wilfordii*), but their status for further clinical trials after preliminary results remains unknown. The structure of some commonly isolated natural products from plants that showed potent anti-inflammatory activities are shown in Figure 1.

### 4.2. Animals—Helminths

Numerous studies using helminths and their products have ameliorated inflammation in various IBD animal models. For instance, in the trinitrobenzene sulfonic acid (TNBS)-induced colitis model, mice infected with *Schistosoma mansoni* or its eggs showed reduced colonic inflammation [55]. Similarly, low-molecular-weight metabolite fractions from somatic extracts and excretory–secretory products (ESPs) of *Ancylostoma caninum* also protect against TNBS-induced colitis in mice by significantly reducing IL-23, TNF, and IL-1β cytokines [56]. Several clinical studies using ESPs from helminths were conducted, among which *Trichuris suis* ova (TSO) stood out as most promising. Recently, the probiotic treatment of UC patients with TSO has completed a phase II clinical trial (NCT03565939); however, the result from this trial is not yet accessible or published [31]. 

Many studies have also examined the possible additive effects of combined therapy using various natural products to identify better therapeutic agents for IBD with fewer side effects. For instance, a study examined the anti-inflammatory potential of *Leiurus quinquestriatus* (LO venom) venom in an acetic acid-induced colitis mice model. In colitic mice, LO venom showed reduced COX-2, IL-22, and TLR-9 expression (Table 3) [57]. The study further assessed the anti-colitic property of LO venom in combination with the IBD drug mesalazine, whereby the combined treatment more significantly protected the colonic tissues of mice [57]. The study did not identify anti-inflammatory components of the crude venom, which is worthwhile to pursue based on promising results. SjDX5-53, a peptide identified from *S. japonicum* eggs, enhanced Treg function, suppressing inflammation in colitis and psoriasis-like models [58]. It mainly induced tolerogenic dendritic cells (tolDCs) via TLR2 signalling, promoting Treg generation and peripheral tolerance [58], suggesting the potential of parasite-derived peptides in treating autoimmune conditions, including IBD (Table 3).

### 4.3. Microbial Sources

Recent research underscores the bidirectional relationship between IBD progression and gut microbiota changes, highlighting the gut microbiome’s dual role in IBD [87,88]. When balanced, the microbiome supports immune regulation, barrier integrity, and overall gut health [89]. For example, gut microbes convert primary bile acids into secondary bile acids, regulate RORγ-expressing Tregs, and metabolize tryptophan, crucial for immune activation and anti-inflammatory responses [90,91,92]. However, in IBD, dysbiosis, an imbalance in the gut microbiota can exacerbate disease progression. The study had shown that microbial species like *Fusobacterium nucleatum* and *Ruminococcus gnavus* were significantly increased in CD compared to controls, while the presence of beneficial microbes such as *Eubacterium rectale* and *Ruminococcus albus*, known for their anti-inflammatory effects, were decreased [93]. This dysbiosis contributes to worsening gut inflammation and the exacerbation of IBD symptoms. In this view, it is essential to understand the connection between IBD and the microbiome to develop novel microbiome-targeted therapies for IBD. 

Microbiota, chiefly probiotic strains, have shown promising anti-inflammatory benefits. Their anti-inflammatory effect is mainly due to their ability to produce short-chain fatty acids (SCFAs), which can restore the population of beneficial gut microbiota while suppressing harmful strains by the protective mucosal layer [84,94]. They also produce anti-inflammatory molecules, chiefly polysaccharides [95]. There are also several studies on the probiotic treatment of IBD using various microbes and their genetically modified species, such as *Escherichia coli* Nissle 1917 [84,94]. Specific probiotic strains like *Lactobacillus plantarum* CKCC1312 and *L. fermentum* CKCC1858 have proven beneficial for UC via promoting mucosal integrity [84]. *Bacteroides thetaiotaomicron* alleviated clinical symptoms of dextran sulfate sodium (DSS)-induced colitis by promoting the differentiation of Treg/Th2 cells and suppressing Th1/Th17 cell development [81]. It significantly boosted FoxP3 expression, demethylated multiple CpG sites in the FoxP3 promoter, and activated AHR, which may have contributed to colitis protection [81]. However, epigenetic FoxP3 regulation by *B. thetaiotaomicron* is implicated with uncertain long-term immune changes; thus, it should be considered cautiously (Table 3). 

Reactive oxygen species (ROS) contribute to intestinal inflammation, implicating antioxidant enzymes like catalase and superoxide dismutase (SOD) in treating IBD. Engineered *Escherichia coli* Nissle 1917 (ECN-pE) overexpressing catalase and SOD, coated with chitosan and sodium alginate via electrostatic assembly, demonstrated enhanced bioavailability in the gastrointestinal tract. In a mouse IBD model, this coated ECN-pE effectively reduced inflammation, repaired epithelial barriers, and positively modulated intestinal microbial communities. These findings suggest a promising approach for using probiotic bacteria to develop living therapeutic proteins for inflammatory intestinal disorders [94]. Minas Frescal cheese containing *Lactococcus lactis* NCDO 2118 probiotic effectively ameliorated DSS-induced colitis in mice by enhancing tight junction, protein gene expression, and modulating cytokine production [84]. Further, it prevented goblet cell damage and reduced inflammatory cell infiltration into the colon mucosa [84]. These findings suggest probiotic functional foods as an adjunct therapy in UC management along with conventional treatments.

Fungi species like *Auricularia polytricha* and *Flammulina velutipes* also showed potential in treating IBD by controlling key signaling pathways, including NF-κB and Keap1/Nrf2, and altering the gut microbiota [96], indicating *A. polytricha* and *F. velutipes* as potential probiotics for gut health. Marine-derived fungi produced unique indole-terpenoids with significant anti-inflammatory activity. Dai et al. [78] isolated 27 compounds from *Penicillium* sp. ZYX-Z-143, including new indole-diterpenoids, penpaxilloids E, schipenindolene A, and paxilline D that inhibited NO production, showing these molecules as novel chemical scaffolds for developing new anti-inflammatory drugs (Table 3).

## 5. Advances in Artificial Intelligence-Guided Drug Discovery for IBD Treatments 

Artificial intelligence (AI) and AI-assisted tools have recently played a vital role in drug discovery. They have leveraged the drug discovery process by increasing efficiency, lowering costs, and improving precision by enabling the prediction of structure–activity and drug–target interactions [97]. AI-driven computer-aided drug design [98] and high-performance algorithms, such as machine learning (ML) and deep learning (DL), quantitative structure–activity relationship (QSAR) modelling, pharmacophore modelling, and de novo drug design have streamlined the drug screening process and helped select promising/hit compounds precisely [99]. For instance, AI was used for developing a protein kinase C (PKC) theta inhibitor (currently in phase 1 clinical trials), and machine learning (ML) was used to integrate many pharmacological characteristics and compute the dosages for the first-in-human trial (FIHT) [100]. 

Artificial intelligence is also applied in analytical chemistry, particularly for phase and baseline corrections and nuclear magnetic resonance (NMR) spectrum analysis while isolating lead compounds from NPs [101]. For instance, NMR machine software such as Bruker’s DL approach and TopSpin software version 4.1.3 achieved human-level accuracy and superior phase and baseline correction for 1D 1H NMR spectra. A DL algorithm can automatically recognise the signal area from NMR spectra, enhancing full automation in analytical chemistry [101]. 

Further, the integration of AI in bioinformatic tools, such as MetaWIBELE (Metagenomic Workflow for Identification of Biologically Enhanced Lysine Export), has assisted in identifying over 340,000 potentially bioactive protein families in active phases of IBD from metagenomic data [102]. The analysis identified possibly contributing targets involving Enterobacteriaceae pilins and VWF-like exoproteins. It also uncovered several other proposed mechanisms of cell–cell communication, such as molybdoproteins and extracellular metabolic chaperones [102]. Other AI-driven tools that help predict and identify metabolites from NPs through clustering analysis include XenoSite’s neural network, DP4-AI, and MS2DeepScore [103,104] (Table 3). Artificial intelligence approaches should be considered cautiously, as many operate as black boxes that do not connect predictions to underlying mechanisms or offer functional explanations for discovered associations, correlations, and recommended decisions. Understanding causal mechanistic insights is essential for clinical applicability in complex and heterogeneous diseases like IBD. Moreover, due to the potential harm of poorly validated models, thorough experimental and clinical validation is crucial before implementing machine learning-based models in clinical practice. From an analytics perspective, it is imperative to prioritize the development of interpretable machine learning models.

AI-based in silico approaches, such as molecular docking and protein–protein interaction studies, have demonstrated that curcumin and epigallocatechin gallate (EGCG) exhibit high binding affinities for the NLRP3 protein within the inflammasome complex, surpassing even that of the selective inhibitor MCC950 [105]. These findings suggest curcumin and EGCG could be promising lead compounds for inflammatory conditions involving NLRP3 inflammasome activation, including IBD [105]. Despite numerous studies revealing the potential anti-inflammatory properties of phenolic acids from plants, the precise anti-inflammatory mechanisms remain unclear. When a study examined the anti-inflammatory properties of chlorogenic acid, rosmarinic acid, and ellagic acid through comprehensive network pharmacology, molecular docking, and dynamic simulations [106], selected phenolic acids suppressed TNF convertase, preventing TNF generation (Table 3).

Molecular docking and ADMET prediction have expedited the drug discovery process by studying the pharmacokinetic properties of drug candidates to identify potentially suitable protein binding sites faster. For instance, the molecular docking investigation of the phytochemical constituents of methanol extract of *Nyctanthes arbortristis* leaves revealed their significant potential for inhibiting both COX-1 and COX-2 enzymes, a finding corroborated by the ADMET analysis. This led to the isolation of abortitristoside A and abortitristoside B, which inhibited COX-2 and COX-1 enzymes [107]. Although NPs remain the primary source of therapeutic leads or scaffolds, the overall drug development process, including clinical trials, continues to be challenged by high attrition rates because of limited funding support due to a lack of patent protection (e.g., for crude NPs) and rigor involved in designing a clinical trial [108]. Additional hurdles that may continue to challenge NP-based drug discovery are lack of accessibility, sustainability, and difficulty synthesising identified drug leads in bulk as required for repeated trials. 

## 6. Limitations and Challenges of Using AI in Drug Discovery from Natural Products

Exploring NPs and their bioactive compounds offers promising drug prospects due to unique mechanisms, low toxicity, and fewer side effects [109]. However, NPs are acknowledged for their multifaceted and varied chemical structures, exhibiting challenges for AI algorithms to model and predict precisely [110]. The distinctive nature of many NPs often results in limited data for AI training, obscuring model simplification. Imprecise or biased data can lead to flawed predictions, impeding the identification of drug candidates [111]. Similarly, AI models, particularly deep learning, often function as black boxes, suggesting predictions without clear justifications, which is challenging in drug discovery, where understanding the mechanism of action is critical [112].

Further, AI predictions necessitate authentication through experimental methods, which can be resource-intensive and time-consuming, limiting their practical application in natural product drug discovery [113]. Additionally, using AI to discover drugs from natural products raises concerns about biopiracy and the equitable distribution of benefits, especially when involving traditional knowledge from indigenous communities [114]. Ensuring ethical AI use in drug discovery, including evading bias in training data and decision-making, is fundamental for fairness and transparency [115]. Hence, interdisciplinary research is crucial for unlocking these natural compounds’ therapeutic potential.

## 7. Conclusions and Future Directions 

Conventional treatments using corticosteroids, aminosalicylates, and immunosuppressants have been fundamental options for IBD patients despite associated side effects. The advent of biologics, including anti-TNFs, JAK inhibitors, and, recently, anti-integrins, has significantly improved IBD treatment outcomes. However, variable efficacy, side effects, and high costs have been major constraints in daily clinical practice. Many new IBD therapeutics are in the pipeline, but they require more clinical validations and safety assessments using more extensive IBD cohort studies. 

Natural products from plants, helminths, and microbes exhibit considerable promise as anti-inflammatory agents for treating IBD, including a few already in early clinical trial phases. (e.g., curcumin and berberine). Helminth excretory/secretory products and probiotic microbial strains, including Lactobacillus species, have also shown efficacy in preclinical and clinical studies. However, they have to pass through more validations for their safety and efficacy before their appearance in the clinical application. The role of the gut microbiome in IBD is rapidly advancing, with probiotics and prebiotics exhibiting potential. However, the exact mechanisms of action remain unclear. Hence, further intensive research is crucial to ascertain specific microbial strains with therapeutic potential, clarify their mechanisms of action, and address the current limited knowledge regarding long-term safety and efficacy.

Artificial intelligence (AI)-assisted technologies, including machine learning and deep learning, have streamlined chemical structure forecasting, synthesis pathway proposals, and drug–target interaction elucidation processes. AI-assisted tools, such as molecular docking and protein–protein interaction studies, have identified promising anti-inflammatory leads such as curcumin and epigallocatechin gallate. However, AI-assisted approaches often need more transparency in their predictions and elucidation of underlying pharmacological mechanisms. Therefore, it is crucial to develop interpretable machine learning models to enhance clinical applicability and ensure the safety and reliability of AI-driven predictions. Thorough experimental and clinical validation of these models is essential before their implementation in clinical practice. While current treatment options have significantly improved IBD patient care, applying AI platforms and interdisciplinary collaborations may further speed up the search for better treatments for IBD. In doing so, we may be able to see at least a few new IBD drugs in the next couple of decades.

## Figures and Tables

**Table 1:** Existing approved drugs for IBD, their brand names, delivery route, and associated side effects.

Types	Drugs	Brand Names	Diseases Target	Delivery Route	Side Effects
Small molecules	Aminosalicylate	Asacol HD, Salofalk, Pentasa, Lialda	Mild to moderate UC	Oral	Burping, constipation, nausea, vomiting, stomach pain/cramping, diarrhoea, dizziness, cold symptoms, back pain, headache, rash, itching, coughing, vomiting, bloody diarrhoea, and rectal bleeding.
	Olsalazine	Dipentum	Induction and maintenance of remission of mild-severe UC	Oral	Diarrhoea, stomach pain, rash, itching, fever, severe muscle aches and weakness, bruising of skin and eyes.
	Sulfasalazine	Azulfidine	Induction of remission of UC	Oral	Nausea, vomiting, stomach upset, loss of appetite, headache, rash, low sperm count in men.
	Balsalazide	Colazal, Giazo	UC and CD	Oral	Headache, fever, diarrhea, nausea, vomiting, abdominal pain, loss of appetite, cramping, and rash.
Corticosteroids	Hydrocortisone	Anucort, Colocort, Cortenema, Cortef, Cortifoam	Helpful for inflammation in the anus, rectum or sigmoid colon in both UC and CD	Rectal	Acne, weight gain, fragility fracture, cataracts, hypertension, diabetes, stretch marks, moon face (rounding of face), insomnia, mood swings, psychosis, weakened bones (osteoporosis), venous thromboembolism (VTE), and increased risks of infections in long-term therapy.
	Prednisone/prednisolone	Deltasone	CD, UC	Oral, IV	
	Methylprednisone	Medrol, Solumedrol	CD, UC	IV, Oral	
	Budesonide	Uceris, Entocort	Active UC and CD with more diffuse disease	Oral; topical enema therapy	
	Beclomethasone dipropionate (BDP)	Clipper	Mild/moderate UC	Oral: enemas, foams or suppositories	Side effects are less than conventional corticosteroids.
Immunomodulators/immunosuppressants	Azathioprine	Azasan, Imuran	CD and UC patients with steroid-resistant or steroid-dependent delay the recurrence of CD after surgical resection	Oral	Pancreatis and suppression of bone marrow and lymphoma.
	6-Mercaptopurine	Purixan	CD and UC patients with steroid-resistant or steroid-dependent delay the recurrence of CD after surgical resection	Oral	Headache, diarrhoea, nausea, vomiting, tiredness, joint pain, mouth sores, rash, fever, and liver inflammation.
	Methotrexate	Trexall	CD with steroid resistance/dependence; CD in children	Oral	Leukopenia, hepatic fibrosis, and hypertensive interstitial pneumonitis.
	Cyclosporine	Neoral, Sandimmune	Severe UC and not responding to glucocorticoid therapy	Oral	Renal insufficiency, hypertension, hepatitis, diabetes, increased cholesterol level, insomnia, and headache.
	Tacrolimus	Prograf	Severe CD	Oral	Diabetes, hepatitis, decreased kidney function, increased cholesterol, insomnia, headache, high blood pressure, swollen gums, seizure, and increased facial hair.
TNF inhibitors	Adalimumab	Humira, Amjevita, Cyltezo, Hyrimoz, Simlandi, Yusimry, Idacio, Imraldi, Amsparity, Hefiya, Hullo	Moderate to severe UC and CD, showing inadequate response to or intolerance to other conventional therapies, including infliximab	SC	Injection site reactions, headaches, rash, nausea, abdominal pain, nausea and vomiting, upper respiratory infections (sinus infections), and muscle pain.
	Golimumab	Simponi	In adults with mild to severe UC, showing inadequate response or intolerance to other medications	SC	Upper respiratory tract infection, reactions at the injection site, and viral infections.
	Infliximab	Avsola, Flixabi, Inflectra, Remicade, Renflexis, Zymfentra, Flixabi, Remsima	Induction and maintenance of remission of moderate-severe UC and CD	IV infusion, SC	Fever, chest pain, respiratory infections, such as sinus infections, sore throat, sweating, nausea, itching, headache, coughing, rash, difficulty breathing, and stomach pain.
	Certolizumab pegol	Cimzia	UC	SC	Upper respiratory infections (flu, cold), rash, and bladder infections.
Anti-IL-12/IL-23 mAb	Ustekinumab	Stelara	Moderate/severe UC and CD	IV infusion, SC	Cold, sore throat or sinus infections, dizziness, headache, diarrhoea, itching, back and joint pain, and muscle fatigue or pain.
Anti-IL-23	Risankizumab	Skyrizi 150 Mg Dose Pack	CD in adults	IV, SC	Cold, sore throat or sinus infections, headache, tiredness, itching, and skin fungal infections.
JAK inhibitors	Tofacitinib	Xeljanz	UC	Oral	Difficulty in breathing or swallowing, rash, hives, swollen face including lips and mouth or swollen hands and feet; common side effects include headache, runny nose, nausea, nasopharyngitis, and joint pain.
	Filgotinib	Jyseleca	UC	Oral	Cold, sore throat, sinus infection, and urinary tract infection; serious side effects might include pneumonia or shingles.
	Upadacitinib	Rinvoq	UC	Oral	Rash, itchy patches on skin [16], swelling lips, tongue or throat, and difficulty breathing or swallowing.
α4β7-integrin mAb	Vedolizumab	Entyvio	Moderate/severe UC and CD	IV infusion	Common cold, headache, joint pain, nausea, and fever.
α4-integrin mAb	Natalizumab	Tysabri	Induction and maintenance of remission of moderate-severe CD	IV infusion	Headache, depression, tiredness, joint pain, urinary tract infections, upper respiratory tract infections, diarrhoea, and stomach pain.
S1P inhibitor	Ozanimod	Zeposia, Zeposia 7-day starter pack, Zeposia starter pack	UC	Oral	Upper respiratory tract infections, headache, urinary tract infections, elevated liver tests, low blood pressure, high blood pressure, and back pain.

UC: ulcerative colitis; CD: Crohn’s disease; IV: intravenous; SC: subcutaneous; JAK: Janus kinase; S1P: sphingosine 1-phosphate. All information in this table are retrieved from DRUGBANK online (https://go.drugbank.com/, accessed on 8 July 2024) and Crohn’s and Colitis Foundation (www.crohnsandcolitisfoundation.org, accessed on 10 July 2024), Crohn’s and Colitis Foundation UK (www.crohnsandcolitis.org.uk, accessed on 28 June 2024), and Crohn’s and Colitis Foundation Australia (www.crohnsandcolitis.org.au, accessed on 25 June 2024).

**Table 3:** Natural product-derived anti-inflammatory agents investigated for treating inflammations and inflammatory disorders.

Species/Source	Anti-Inflammatory Compounds/Products	Model/Cell	The Main Effect on Inflammation	Ref.
Plants				
*Alhagi pseudalhagi* (M.Bieb.) Desv. ex Wangerin	Alhagi honey polysaccharide (AHPN50-1a)	DSS-induced colitis mice	Downregulated IL-1β, IL-6, and TNF expression in colon tissue Restored microbiota diversity and increased concentrations of short-chain fatty acids (SCFAs) produced by gut microbiota	[59]
*Alpinia zerumbet* var.	Homogeneous polysaccharide (AZP-2)	stimulated RAW264.7 cell	Inhibited NO, ROS, and increased IL-10 production Regulates the NF-κB signaling pathway	[60]
*Andrographis paniculata* (Burm.f.) Wall. ex Nees	Andrographolide	LPS-stimulated RAW264.7 cells	Degradation of MK2 concentration Inhibit TNF, MCP-1	[61]
*Curcuma longa* L.	Curcumin	DSS-induced colitis mice	Inhibited IL-1β, IL-2, IL-6, IL-9, and IL-17A production	[62]
*Hydrastis canadensis* L.	Berberine (Berberine chloride)	DSS-induced colitis in rats	Increased TNF, IL-1β and IL-6 Decreased IL-10	[63]
*Hypericum sampsonii* Hance	Hypersampsonone H	LPS-induced RAW264.7 cells	Suppressed NO production Inhibited COX-2 and iNOS, IL-6, TNF and IL-10 expression	[64]
*Magnolia officinalis* Rehder & E.H.Wilson	Honokiol	DSS-induced colitis mice (C57BL/6J mice)	Decreased TNF, IL-6, IL-1β, and IFN-γ Increased PPAR-γ expression Downregulated TLR4, NF-κB signaling pathway	[65]
*Nelumbo nucifera* Gaertn	Nuciferine	LPS-induced RAW 264.7 cells	Reduced the expression of iNOS, IL-1β, IL-18, and TNF. Disrupted the activation of MAPK, NF-κB, and NLRP3 signaling pathways	[66]
*Piper methysticum G.* Forst	Flavokawain B	C57BL/6 J mice	Inhibited NF-κB signaling pathway	[67]
Plants	Coumaric acid and syringic acid	Acetic acid-induced colitis mice	Downregulated TNF and IL-1β and upregulate the Nrf2/HO-1 pathway	[68]
*Polygoni multiflori* Radix	2,3,5,4′-Tetrahydroxystilbene-2-O-β-D-glucoside	DSS-induced colitis mice (BALB/c)	Inhibited TNF-α, IL-1β, and IL-6 and IL-10 expression level Increased the abundances of *Firmicutes* and *Bacteroidetes* Improved the homeostasis of the gut microbiota composition	[69]
*Poncirus trifoliata* (L.) Raf	Poncirin, naringin, imperatorin, and phellopterin	LPS-induced RAW 264.7 cells	Inhibited NO and iNOS production	[70]
*Strongylocentrotus nudus* (A. Agassiz)	Polysaccharides from egg	DSS-induced acute ulcerative colitis mice (C57BL/6 J mice)	Inhibited IL-6, IL-1β, TNF production Suppressed Th17 and increased Treg cells production	[71]
*Tetrastigma hemsleyanum* Diels & Gilg	Nicotiflorin	DSS-induced colitis mice (C57BL/6 mice)	Inhibited the activation of NF-κB and NLRP3 inflammasomes.	[72]
*Tubocapsicum anomalum* (Franch. & Sav.) Makino	Tubocapsanolide A	DSS-induced colitis mice (C57BL/6 mice)	Suppression of INF-γ, IL-6, TNF, and IL-6 levels in serum and colonic tissue	[73]
Fungi				
*Phellinus baumii*	Heteropolysaccharide (SHPS-1)	LPS- LPS-stimulated macrophage RAW 264.7 cells	Downregulated iNOS and TNF level Upregulated IL-10 expression	[74]
*Antrodia cinnamomea*	Antcin-H	DSS-induced colitis mice (C57BL/6JNal mice)	Inhibits colonic expression of NLRP3, ASC, active caspase-1, IL-1β, IL-6, TNF	[75]
*Ganoderma lucidum*	Baoslingzhine K	LPS-stimulated RAW264.7 cells	Inhibited protein expression of iNOS and COX	[76]
*Porphyra haitanensis*	Oligosaccharides (PHO)	LPS-induced IEC-6 cells	Upregulated ZO-1, claudin-1, and occluding Downregulated oNF-κB p50 and NF-κB p65 pathways, Inhibited the TLR4/NF-κB pathway	[77]
*Penicillium* sp. ZYX-Z-143-fgi	Penpaxilloids E, schipenindolene A, paxilline D	LPS- LPS-stimulated RAW264.7 macrophages	Suppressed NO production	[78]
Microbial				
*Actinoalloteichus* *Cyanogriseus*	Cyanogramide	THP-1 cells and a Caco-2/THP-1	Inhibited IL-6 secretion	[79]
*Bacteroides ovatus*	indole-3-acetic acid	TNBS-induced colitis mice	Upregulated IL-22 expression	[80]
*Bacteroides thetaiotaomicron*	IAA (Indole-3-Acetic Acid) and IPA (Indole-3-Propionic Acid)	DSS-induced colitis mice	Regulated the Th17/Treg balance and restore immune homeostasis	[81]
*Lactococcus lactis* NCDO 2118	Minas Frescal Cheese	DSS-induced colitis mice	Increased gene expression of tight junctions’ proteins zo-1, zo-2, ocln, and cln-1 in the colon and increase IL-10 release	[82]
*Lacticaseibacillus rhamnosus*	L. rhamnosus IDCC 3201	DSS-induced RAW 264.7 macrophages	Downregulated TNF, IL-6, NO, iNOS) and COX-2 expression levels	[83]
*Lactobacillus fermentum* and *L. plantarum*	*Lactobacillus fermentum* CKCC1858 and *Lactobacillus plantarum* CKCC1312	DSS-induced colitis mice	Increased the level of mucin-2, zonula occludens-1 and interleukin-10 Decreased the levels of IL-1β, IL-17A, IFN-γ, iIL-6 and TNF	[84]
*Serratia sp.*	Prodigiosin	DSS-induced colitis mice	Suppressed IL-1β, IL-6 and IL-10 expression Upregulated junction protein Claudin-1, Occludin and ZO-1	[85]
Animals				
*Rhopilema* *esculentum* Kishinouye	Skin polysaccharides	DSS-induced colitis mice (C57BL/6J)	Downregulated MPO, NO level Upregulated TNF, IL-6, L-1β level Upregulated Occludin, ZO-1, Muc2 level	[86]
*Leiurus quinquestriatus* (Ehrenberg)	L.Q venom	Albino CD-1 mice	Downregulated TREM, NO, MPO level in sera and MMP-9, caspase-3, NO, MPO in colonic tissue	[57]
Parasite				
*Schistosoma japonicum*	Small three kDa peptide (SjDX5-53)	C57BL/6 mice	Induce Tregs and inhibit T-helper (Th1/Th17)	[58]

COX-1: Cyclooxygenase-1; COX-2: Cyclooxygenase-2; DSS: Dextran sulfate sodium: IFN-γ: Interferon-gamma: IL: Interleukin; LPS: Lipopolysaccharide; MAPK: Mitogen-activated protein kinases; MCP-1: Monocyte Chemoattractant Protein-1; MPO: Myeloperoxidase; NF-κB: Nuclear Factor Kappa Beta; NO: Nitric oxide; PGE2: Prostaglandin 2; PPAR-γ: Peroxisome Proliferator-Activated Receptor Gamma; RAW: Ralph and William’s cell line; ROS: Reactive oxygen species; TLR4: Toll-like receptor 4; TNF: Tumor necrosis factor.

## Data Availability

Data sharing is not applicable.
